# Brain-computer interfaces: an engineering black-box swindle or a lone advance guided by deep learning

**DOI:** 10.3389/fnins.2026.1783020

**Published:** 2026-04-17

**Authors:** Zhengbo Yuan, Zhongjie Shi, Zhanxiang Wang

**Affiliations:** 1 Department of Neurosurgery, The First Affiliated Hospital of Xiamen University, School of Medicine, Xiamen University, Xiamen, Fujian, China; 2 Xiamen Neurosurgical Quality Control Center, Xiamen, Fujian, China; 3 National Institute for Data Science in Health and Medicine, Xiamen University, Xiamen, Fujian, China; 4 Department of Neurosurgery and Department of Neuroscience, Fujian Key Laboratory of Brain Tumors Diagnosis and Precision Treatment, The First Affiliated Hospital of Xiamen University, School of Medicine, Xiamen University, Xiamen, Fujian, China

**Keywords:** brain-computer interface (BCI), clinical translation, co-adaptation, neural decoding, neural manifolds, neuroprosthetics

## 1 Introduction

Brain-computer interfaces (BCIs) have evolved from speculative science fiction to tangible neurotechnology, with clinical systems now enabling paralyzed individuals to control assistive devices. Yet a persistent question remains: do BCIs truly “understand” neural intent, or do they merely fit statistical models to observable signals? This debate often polarizes into two camps: one dismissing BCIs as “engineering black-box swindles,” and the other heralding them as “lone advances guided by deep learning.” Here, we argue that both extremes overlook the essence of modern Brain-computer interface (BCI) systems—bidirectional co-adaptation between brain and machine.

This opinion article examines the interpretive and methodological challenges in BCI research while also translating them into practical priorities for clinically meaningful, stable, and scalable BCI development. Specifically, we focus on three linked themes: (i) why “black-box” decoding can be a principled approximation problem rather than a philosophical failure; (ii) how recording constraints and low-dimensional structure jointly shape what BCIs can infer and control; and (iii) why non-stationarity and co-adaptation should be treated as central design variables rather than afterthoughts. Building on these themes, we further outline concrete future directions that may help move the field from conceptual debate toward constructive clinical and technological advancement.

## 2 The dimensionality reduction dilemma: from black box to functional approximation

A common critique of BCIs is their reliance on “black-box” models—such as Kalman filters or deep neural networks—that perform dimensionality reduction on high-dimensional neural data. Detractors label this as overfitting or “hard-coding.” However, we reframe this process as necessary function approximation. Consider the following formulation:

- Teacher Model: the brain (unknown architecture, unknown weights, highly nonlinear, and state-dependent).
- Student Model: the BCI decoder (for example, a Kalman filter or a transformer-based model).
- Input: recorded neural activity (for example, spikes or local field potentials).
- Target: task-relevant variables such as cursor velocity, prosthetic-arm kinematics, or speech-related features.

According to the Universal Approximation Theorem, as long as the student model (decoder) has sufficient capacity and enough observed features (neurons), one can fit the teacher model's (brain's) projection under a specific task. This holds mathematically.

In practice, dimensionality reduction is not a mechanistic shortcut but a computational strategy to extract task-relevant information from noisy, high-dimensional inputs. For instance, the Unscented Kalman Filter improves upon linear models by capturing nonlinear neural dynamics (Li et al., 2009, 2016), while deep learning architectures enable robust feature learning from large-scale neural recordings. These approaches do not claim to reveal the brain's internal logic; rather, they establish usable mappings that facilitate real-time control under hardware constraints.

We argue that mapping complex neural activity into a low-dimensional space currently enables real-time decoding of electrophysiological signals for external device control under constrained computational resources (Tankus et al., 2012; Cai et al., 2025). In this context, dimensionality reduction should not be viewed as a violent simplification of the brain, but as a principled way to extract task-relevant structure from high-dimensional and noisy activity. Describing the closed-loop BCI paradigm as “engineering hard-coding” and “forced dimensionality-reduction fitting of chaotic signals” is an oversimplification; rather, it reflects a pragmatic scientific strategy for extracting task-relevant structure from complex neural activity. The key scientific question is therefore not whether a decoder fully recovers the brain's entire internal mechanism, but whether it captures sufficient structure to support reliable and clinically meaningful control. Future work in this area should move beyond the binary opposition between “interpretability” and “performance,” and instead ask which levels of representation are necessary for robust decoding, generalization across sessions, and practical bedside usability.

## 3 Neural drift and adaptive decoding: the dynamic brain meets dynamic models

A given neuron may contribute to different task variables across adjacent moments and behavioral contexts. Modern neuroscience emphasizes mixed selectivity, namely that single neurons can encode multiple task variables in a context-dependent manner, thereby expanding representational capacity (Tye et al., 2024). For BCI systems, this implies that the mapping between recorded activity and behavioral variables is not strictly fixed. Across minutes, days, and sessions, changes in neural recruitment, recording conditions, or latent population structure can produce neural drift, causing a previously effective decoder to lose accuracy and forcing repeated recalibration. The key challenge, therefore, is not to assume a static brain, but to design dynamic models that can track non-stationarity while preserving stable control.

Critically, drift adaptation does not require full observation of internal brain states. Instead, it leverages the geometric stability of neural manifolds—low-dimensional subspaces that capture task-relevant neural variability. By monitoring manifold structure, decoders can infer drift direction and adjust without explicit supervision. This approach aligns with the principle that effective BCI control depends not on reading “all neurons,” (Cunningham and Yu, 2014) but on extracting consistent computational motifs from population activity.

Importantly, a low-dimensional manifold does not mean that we can simply reduce the number of recorded signals, because it only indicates that activity does not fill the entire ambient space, while any given activation unit may still be critically engaged. For example, the raw image space is 256 × 256 × 3, which contains vast amounts of meaningless content (e.g., pure noise images); thus, natural images occupy a low-dimensional manifold within that space. Yet this manifold does not mean that you can reduce the number of pixels, because every pixel in a given photograph can be meaningful. What may be reduced are redundant degrees of freedom: neurons within a region may be functionally convergent and perform similar roles, yet the finer the parcellation, the richer the representational content we can access. Analogously, in image recognition, a low-resolution picture may still reveal an animal's color, yet make it difficult to distinguish its specific category.

Finally, it is worth separating interpretive models from operational decoders. The Population Vector (PV) model is historically important for understanding directional tuning, but it is not a general “principle” that explains modern closed-loop decoding (Mahan and Georgopoulos, 2013). Contemporary online decoding has relied on state-space models and adaptive extensions, and recent work has explored distribution-shift–aware learning (including adversarial approaches) to extend decoding accuracy over time (Ma et al., 2023). Efficient formulations such as steady-state Kalman filtering further illustrate how algorithmic choices can trade off performance, stability, and computational burden in real systems (Malik et al., 2011). A mechanistic “principle” is rarely a single model; it is a set of constraints that remains useful across tasks, recordings, and adaptation regimes. Accordingly, future decoder development should prioritize adaptive and uncertainty-aware models that reduce recalibration burden, preserve performance across sessions, and explicitly benchmark robustness to drift in clinically relevant settings.

## 4 From mechanism to function: the co-adaptive BCI paradigm

Some argue that BCIs must first “fully understand the brain” to achieve refined control. This view conflates mechanistic explanation with functional utility. Connectomics and circuit-level insights are invaluable, but BCI success does not presuppose complete neural mapping. Instead, practical systems rely on co-adaptive learning: users learn to modulate recorded neural populations, while decoders adapt to individual neural patterns.

When first encountering the neural-manifold framework, some readers may assume that neuronal activity unfolds in a fully high-dimensional space and that current electrodes capture only a low-dimensional slice of that activity. However, this interpretation is incomplete. Although the overall neural system is extremely high-dimensional, many task-related activity patterns evolve on relatively low-dimensional manifolds. But this is not the case; although neuronal dimensions are extremely high, many task-related activities often lie on a relatively low-dimensional subspace/manifold. In other words, the “dance” originally occurs on a low-dimensional structure. Even in a brain containing billions of neurons, specific behaviors such as arm movement may be governed by a limited number of latent factors because relevant neural populations covary strongly during task execution.

Evidence from clinical BCI studies (e.g., BrainGate) shows that users develop stable cortical maps for prosthetic control, akin to motor skill acquisition (Hochberg et al., 2006; Willsey et al., 2025). This process is not a passive accommodation to “clumsy algorithms” but an active formation of alternative functional pathways. Thus, BCIs are best understood as bidirectional interfaces that exploit neural plasticity to build new, controllable outputs from observable signals.

Closed-loop BCI research has therefore increasingly emphasized co-adaptation. Decoder updates and user learning jointly shape neural activity and performance, rather than placing the full burden on either side. Closed-loop decoder adaptation (CLDA) has been shown to shape neural plasticity and improve skillful neuroprosthetic control (Orsborn et al., 2014), and BCI paradigms have been used as tools to parse learning in networks (Orsborn and Pesaran, 2017). In humans, these principles support high-performance neuroprosthetic control (Collinger et al., 2013) and multi-dimensional anthropomorphic arm control, while also exposing practical limitations and strategies to mitigate them (Wodlinger et al., 2015). At the neural level, learning under a fixed decoder can lead to the emergence of stable cortical activity patterns that function like a callable skill (Ganguly and Carmena, 2009). Taken together, the field is moving toward a view of BCI not as “the brain accommodating a clumsy algorithm” or “the machine fully understanding the brain,” but as the construction of a stable coupled system. Future closed-loop BCI studies should therefore jointly optimize decoder updating, user training, feedback design, and shared autonomy, rather than treating these factors as isolated sources of variance.

## 5 Discussion

BCIs are not mind-reading devices, and they are not merely curve-fitting either. As a scientific object, a modern BCI is a coupled dynamical system in which a neural population, a decoder, and an effector co-adapt through feedback. Treating decoding as task-level function approximation clarifies both the power and the limits of the approach: a decoder can learn a useful projection without revealing the full causal circuit that generated the activity. That is an acceptable starting point—provided we explicitly evaluate stability, generalization, and the consequences of adaptation.

The question of whether BCIs “understand” the brain is perhaps misleading. It presupposes a dichotomy between mechanistic transparency and functional utility. We propose that modern BCIs operate in a middle ground: they use engineering-driven approximations to decode neural signals, while leveraging neuroscientific principles—such as population coding, manifold geometry, and plasticity—to improve robustness and adaptability. Current brain-machine systems can stably decode intention- or movement-related states from recordable neural activity to control external effectors, thereby establishing alternative functional pathways. From the outset, brain-machine research has been framed as a problem of decoding usable signals to control devices, rather than as a requirement to solve the entire brain before intervention (Andersen et al., 2004). In this sense, current brain-machine systems are better understood as constructing a new controllable closed loop from observable signals than as requiring full reconstruction of the original biological pathways before control can be achieved. Contemporary views of motor control likewise emphasize distributed, parallel, and interacting loops rather than a strictly serial chain of computation. Information transmission in the human brain does not have to operate in a manner where the “previous level must fully compute before passing it to the next” (Andersen and Cui, 2009; Churchland and Shenoy, 2024).

### 5.1 Future research directions and practical recommendations

To translate the above conceptual issues into a constructive research agenda, we suggest four practical priorities for the next phase of BCI research. First, BCI performance should be judged not only by offline decoding accuracy, but also by cross-session stability, recalibration burden, task generalization, safety, and clinically meaningful functional gain. Second, neural drift should be treated as a design target rather than merely a nuisance variable; adaptive and uncertainty-aware dynamic decoders should be developed to reduce frequent retraining and preserve control under non-stationary neural conditions (Ma et al., 2023; Malik et al., 2011). Third, co-adaptation should be operationalized as a research program in which decoder updating, user learning, feedback design, and shared autonomy are jointly optimized, rather than evaluated separately (Orsborn et al., 2014; Orsborn and Pesaran, 2017). Fourth, progress in algorithms must be matched by progress in interfaces, including multimodal sensing, improved electrode biocompatibility, and recording strategies that better balance invasiveness, stability, and signal richness. In this view, current BCI challenges are not closed roads but tractable design constraints that should redirect the field from debates about “black boxes” toward system validation—-namely, whether a BCI provides reliable, generalizable, and clinically meaningful control.

In conclusion, BCIs are neither swindles nor purely data-driven triumphs. BCIs are evolving neurotechnologies that succeed by coupling engineering pragmatism with neuroscientific insight. The field should therefore move beyond asking whether BCIs completely “understand” the brain and instead focus on building systems that are stable, adaptive, safe, and clinically meaningful. By embracing co-adaptation as a core principle and by pursuing the practical priorities outlined above, BCI research can advance toward more intuitive, reliable, and accessible brain-machine communication.
